# Vascular Dysfunction Induced by Mercury Exposure

**DOI:** 10.3390/ijms20102435

**Published:** 2019-05-16

**Authors:** Tetsuya Takahashi, Takayoshi Shimohata

**Affiliations:** 1 Department of Neurology, National Hospital Organization Nishiniigata Chuo Hospital, Niigata 950-2085, Japan; 2 Department of Neurology, Gifu University Graduate School of Medicine, Gifu 501-1194, Japan

**Keywords:** blood–brain barrier, methylmercury, vascular endothelial growth factor, l-type amino acid transporter 1

## Abstract

Methylmercury (MeHg) causes severe damage to the central nervous system, and there is increasing evidence of the association between MeHg exposure and vascular dysfunction, hemorrhage, and edema in the brain, but not in other organs of patients with acute MeHg intoxication. These observations suggest that MeHg possibly causes blood–brain barrier (BBB) damage. MeHg penetrates the BBB into the brain parenchyma via active transport systems, mainly the l-type amino acid transporter 1, on endothelial cell membranes. Recently, exposure to mercury has significantly increased. Numerous reports suggest that long-term low-level MeHg exposure can impair endothelial function and increase the risks of cardiovascular disease. The most widely reported mechanism of MeHg toxicity is oxidative stress and related pathways, such as neuroinflammation. BBB dysfunction has been suggested by both in vitro and in vivo models of MeHg intoxication. Therapy targeted at both maintaining the BBB and suppressing oxidative stress may represent a promising therapeutic strategy for MeHg intoxication. This paper reviews studies on the relationship between MeHg exposure and vascular dysfunction, with a special emphasis on the BBB.

## 1. Introduction

Mercury is one of the most toxic heavy metals. Mercury exists in the environment in three forms: elemental, inorganic, and organic. Although elemental and inorganic mercury can cause human health problems, exposure to these forms is generally limited to certain subpopulations, e.g., people working in the manufacturing of mercury-containing drugs or dentists using dental amalgam. Methylmercury (MeHg) is the most common and toxic form of organic mercury. In humans, mercury is readily absorbed into the body, which has no active excretion system for this element [1]. MeHg severely damages the central nervous system (CNS), and increasing evidence shows an association between MeHg exposure and vascular dysfunction.

In the 1950s, industrial waste containing MeHg caused severe poisoning, the so-called "Minamata disease" and “Niigata Minamata disease”, in Japan [2,3,4,5,6]. MeHg bioaccumulates through the food chain; thus, people who ingested highly contaminated fish and shellfish were affected. Clinical manifestations of these diseases include cerebellar ataxia, concentric constriction of the visual field, and sensory and auditory disturbances. The specific symptoms depend on the lesions induced by MeHg, which includes damage to the cerebellum and occipital lobes. However, the underlying mechanism of MeHg-induced selective tissue vulnerability remains to be elucidated. Post-mortem pathological studies of the abovementioned diseases showed petechial hemorrhage and edema in the brains of patients with severe disability [7]. This suggests that MeHg possibly causes blood–brain barrier (BBB) damage. Numerous studies have shown that long-term exposure to small amounts of mercury affects endothelial cells and may be associated with an increased risk of cardiovascular diseases [8,9]. MeHg is found not only in industrial waste, but also occurs in nature, where it is formed through microbial methylation of mercury. Although extensive artificial MeHg pollution has not occurred recently, emissions of mercury into the atmosphere from human activities such as gold mining or fossil fuel burning are increasing and are regarded as a public health concern [8,10]. Mercury may also have adverse effects on child development and is considered one of the top 10 chemicals of major public health concern by the World Health Organization [11].

This paper reviews studies on the relationship between MeHg exposure and vascular dysfunction, with a special emphasis on the BBB to raise awareness of the importance of BBB dysfunction in MeHg-induced toxicity.

## 2. Systemic Vascular Effects of MeHg Intoxication

In contrast to the abovementioned MeHg intoxication due to exposure to relatively high amounts, most studies have investigated cardiovascular effects caused by long-term low-dose MeHg exposure. One major source of continuous MeHg exposure is fish, especially large carnivorous fish such as tuna, which bioaccumulate MeHg at high concentrations [12]. Another source is grain crops grown on contaminated soil near gold mines [13] or coal-fired power plants [14] in developing countries. Since the 1990s, numerous reports have suggested that long-term consumption of these contaminated foods may cause hypertension or be associated with an increased risk of ischemic heart disease [8]. In the area around Minamata bay, where the Minamata disease outbreak occurred, the incidence of hypertension increased during the period of MeHg exposure and subsequently decreased [15]. Numerous studies have shown a positive correlation between mercury exposure and hypertension or cardiovascular events [16,17,18,19].

Diverse molecular mechanisms underlie these cardiovascular risks, and the most widely reported is damage to vascular endothelial cells induced by oxidative stress produced in response to MeHg. It is not clear how MeHg raises the production of oxidative agents, but several studies have shown that MeHg induces both a decrease in antioxidant activity and an increase in oxidative stress. Oxidative stress via various pathways induces endothelial inflammation, resulting in endothelial dysfunction [20,21]. MeHg has a high affinity for sulfhydryl groups, including glutathione, and thus can bind and inhibit several antioxidants in the blood [22,23,24]. Decreases in glutathione levels and glutathione-related enzymes have been observed in animal [25,26] and human [27,28,29] studies. MeHg also has a high affinity for selenium compounds, and this leads to decreased antioxidant activity of selenium-containing enzymes, such as glutathione peroxidase [30,31]. Polymorphisms in antioxidant genes, such as glutathione-related genes, in mercury-exposed regions have been associated with methylmercury retention and risk of myocardial infarction [32,33,34]. In addition, MeHg is thought to induce oxidative agents independently of its effects on antioxidants [35,36]. Increased oxidative agents induce mitochondrial dysfunction through mitochondrial DNA damage and respiratory changes and activate a positive feedback loop, resulting in the generation of more oxidants. 

Endothelial cells are vulnerable to oxidative stress, which when increased by MeHg causes endothelial dysfunction, leading to the development of atherosclerosis, thrombosis, vasospasm, and inflammation [37,38]. Consequently, it is associated with risk of atherothrombotic diseases, such as hypertension, cerebrovascular diseases, renal dysfunction, and acute myocardial infarction [39,40,41,42,43]. In addition, MeHg reduces endothelial cell formation and migration [44,45], stimulates vascular smooth muscle cell proliferation [46], and causes platelet activation [47,48] or increases the activity of several coagulation factors, such as factor XIII [49], resulting in a tendency toward hypercoagulation. These effects concomitantly increase the risk of atherosclerotic vascular disease.

## 3. CNS Effects of MeHg Intoxication

The CNS is the organ system most susceptible to MeHg toxicity, and CNS selectivity is mediated by the efficient transport of MeHg into the brain. MeHg interacts with and binds to sulfhydryl group molecules, such as l-cysteine. The MeHg–l-cysteine complex is a substrate for the l-type amino acid transporter 1 (LAT1), which actively transports MeHg across membranes [50]. LAT1 is expressed in various types of brain cells, including neurons, and tissue distribution studies have shown that LAT1 is expressed at higher levels in brain tissue than in other organs [51]. In vitro studies have shown increased MeHg uptake in cells overexpressing LAT1, whereas the uptake of MeHg–l-cysteine and MeHg cytotoxicity were attenuated after LAT1 knockdown [52].

Several mechanisms underlie the toxicity of MeHg towards neuronal cells, including its affinity for sulfhydryl groups. MeHg interacts with sulfhydryl groups of tubulin in microtubules [53,54], which are important in CNS development, and thus inhibits their organization. MeHg also affects gamma-aminobutyric acid receptors by its action on sulfhydryl groups [55] and modifies the *N*-methyl-d-aspartate receptor system [56,57]. However, the most extensively studied and confirmed mechanism is related to the oxidative stress induced by MeHg [58,59,60], which is one of the reasons why MeHg primarily affects the brain. The brain consumes oxygen at a high rate and is a rich source of fatty acids and metals, and therefore is more prone to oxidative stress. MeHg decreases cellular antioxidant activity by directly interacting with antioxidants or selenium. Neurons have more limited defense functions against oxidative stress than other brain cells, such as astrocytes. Therefore, neurons are vulnerable to oxidative stress and are considered to be impaired by oxidative stress in various diseases, including cerebrovascular disease [61,62], Parkinson’s disease [63,64,65], Alzheimer’s disease [66,67,68], amyotrophic lateral sclerosis [69,70,71], and other neurodegenerative diseases. Numerous studies have shown that the presence of antioxidant or selenium reduces MeHg toxicity both in vivo and in vitro [72,73,74,75].

There have been several reports on the relationship between MeHg and immunoreactions. Proinflammatory cytokines, such as interleukin-6 and tumor necrosis factor-alpha, are highly expressed in MeHg intoxication models [76,77,78]. However, these reactions are commonly seen in tissues exposed to oxidative stress; therefore, it is possible that these immune responses are responses to oxidative stress. These cytokines stimulate microglia, leading to changes in microglial polarization and subsequent induction of cellular damage [79].

## 4. MeHg Causes BBB Dysfunction In Vitro

Endothelial cells are also damaged by oxidative stress. Hemorrhage and edema are seen in the brains but not in the systemic vessels of patients with MeHg intoxication. MeHg causes dysfunction of systemic vessels and increased risk of ischemic heart disease or hypertension, but there are only a few reports on associations between MeHg exposure and increased risk of cerebrovascular diseases. These phenotypic differences in vascular dysfunction between the brain and systemic vessels may be caused by structural differences, e.g., vessels with or without BBB. The BBB is composed of brain microvascular endothelial cells, pericytes, astrocytes, and a noncellular component, the basement membrane. The differential vascular reactivity may be due to differences in the reactivity of cells that constitute the BBB other than endothelial cells, namely pericytes and astrocytes. Pericytes surround the endothelial cells and their contraction and relaxation regulate microcirculation [80]. Pericytes also play an important role in vascular remodeling; for example, they secrete extracellular matrix proteins to support vascular integrity [81,82,83]. Astrocytes are attached to the basement membrane of vessels and neurons by end feet processes that extend from the cell body. Astrocytes are thought to maintain cellular homeostasis by regulating water, ion, and amino acid balances through interaction with neurons and endothelial cells [84,85,86]. Astrocytes have high levels of antioxidants, including glutathione-related enzymes, which may be suppressed by MeHg intoxication [87,88].

The BBB controls the selective delivery of molecules from the blood to the brain parenchyma. Disruption of the BBB allows inappropriate molecules or cells to penetrate the brain, causing serious damage. It has been hypothesized that when inflammatory cells and cytokines infiltrate the brain parenchyma, uncontrolled inflammatory responses might occur and injure the brain tissue around the vessels. This has been considered a potential pathological mechanism in several diseases, such as cerebral ischemia, viral encephalitis, and traumatic brain injury [89,90,91]. Dysfunction of any of the three cell types of the BBB, which play different critical roles in maintaining vascular integrity, can impair the barrier function. MeHg passes the BBB mainly via LAT1 on endothelial cells [1]. Similar to all other endothelial cells in the body, brain endothelial cells are impaired by MeHg-induced oxidative stress. MeHg inhibits the proliferation of endothelial cells [44,92] by reducing the expression of fibroblast growth factor-2 [93]. The expression of vascular endothelial growth factor (VEGF) and VEGF receptor-1/-2 in endothelial cells is upregulated after MeHg exposure [94]. VEGF, which is important for endothelial cell migration, proliferation, and maturation, induces hyperpermeability of vessels, resulting in vascular leakage and edema [95].

Pericytes also express LAT1, and they are relatively more vulnerable to MeHg than endothelial cells because they express lower amounts of protective sulfhydryl-containing molecules [96]. Pericyte contraction is induced by oxidative stress, leading to BBB breakdown and blockage of the microvascular blood flow [97]. Pericytes secrete hyaluronan, one of the extracellular matrix components. Hyaluronan has strong water retention ability and regulates the water content of the extracellular fluid. After MeHg exposure, hyaluronan secretion from pericytes is increased [98], which might be one of the causes of brain edema seen in the brains of patients with MeHg intoxication.

Astrocytes have high antioxidant capacity and protect neurons from oxidative stress. However, astrocytes are also subject to oxidative stress as they are vulnerable to oxidative stress when cultured with neurons. Astrocytes show higher uptake of MeHg and glutamate in coculture with neurons than in monoculture, indicating that they protect neurons from toxicity [99]. In addition, aquaporin-4 (AQP4) inhibition by MeHg may contribute to dysfunction of astrocytes. AQP4 is a member of the aquaporin family of water channel proteins. It is distributed predominantly in the mammalian brain and is specifically expressed in the end feet of astrocytes [100]. Mercury is a strong inhibitor of AQP4 [101]. AQP4 regulates the cerebral water balance and is involved in edema development induced by several neurological diseases, such as cerebral ischemia, subarachnoid hemorrhage [102,103], acute water intoxication [104], and traumatic brain injury [105,106]. Although the exact mechanism underlying edema formation associated with AQP4 is unclear, MeHg possibly affects the water-regulating function of astrocytes.

A study in an in vitro BBB model using primary porcine brain capillary endothelial cells revealed that both organic and inorganic mercury induced hyperpermeability of the BBB [107]. While in vitro experiments suggest that BBB disruption occurs if one of the three types of cells of the BBB is impaired, there are no reports that directly show that the dysfunction of astrocytes or pericytes induces BBB disruption.

## 5. MeHg Causes BBB Dysfunction In Vivo

**Figure 1:**
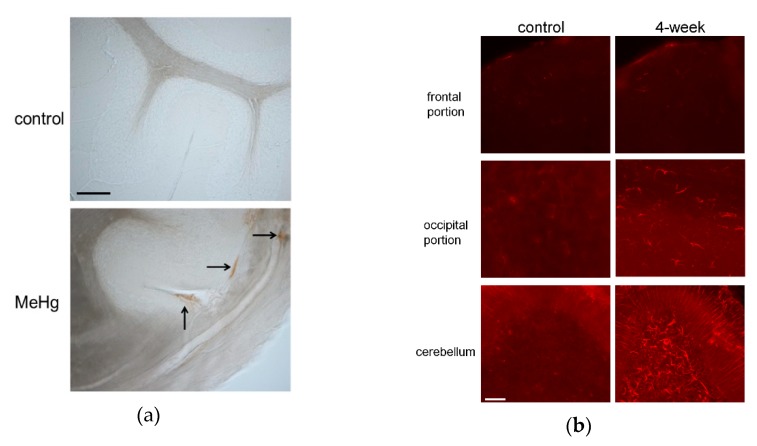
(**a**) Immunoglobulin G (IgG) extravasation in rat cerebellum exposed to methylmercury (MeHg). Rat cerebellum sections from control and 4-week exposure groups (upper and lower panels, respectively) were stained with antibody against rat IgG. Vascular hyperpermeability was evaluated by immunostaining intrinsic IgG outside vessels in control and 4-week exposure groups. Arrows indicate IgG extravasation in the 4-week MeHg exposure group. No IgG staining was detected outside vessels in the control group. Scale bar: 25 μm. (**b**) Vascular endothelial growth factor (VEGF) expression associated with MeHg exposure. Immunohistochemical staining was performed using rabbit anti-VEGF antibody to detect VEGF expression in the frontal and occipital regions and cerebellum of rats in control and 4-week MeHg exposure groups (left and right panels, respectively). Scale bar: 50 μm.

It remains to be confirmed whether MeHg induces BBB disruption and worsens tissue damage in vivo. A recent study demonstrated that MeHg induces VEGF expression in cultured endothelial cells. Increased VEGF expression results in hyperpermeability not only of systemic vessels, but also of the BBB. BBB leakage leads to hemorrhage, edema, and microcirculation failure in a number of diseases [95]. Therefore, we investigated whether MeHg causes BBB damage by inducing VEGF expression in vivo using a rat model of subacute MeHg intoxication [108]. The model was established by exposing the rats to 20-ppm MeHg for up to four weeks, which caused severe pathological changes in the cerebellum, although there were no significant differences in mercury content among the different brain regions. BBB damage in the cerebellum after MeHg exposure was examined based on extravasation of endogenous immunoglobulin G (IgG). The passage of IgG into the brain is usually very limited, and immunohistochemical staining revealed that IgG was detected in the brain parenchyma only in the MeHg exposure group (Figure 1a). In addition, the expression of rat endothelial cell antigen-1 (RECA-1), an endothelial cell marker, was reduced in the MeHg exposure group. Next, we examined the effect of MeHg exposure on VEGF expression. VEGF expression was markedly increased in the cerebellum and mildly in the occipital lobe following MeHg exposure (Figure 1b). We also investigated the cellular localization of VEGF using antibodies against the astrocyte marker glial fibrillary acidic protein (GFAP) and RECA-1. VEGF was expressed on the outer side of RECA-1-positive endothelial cells, and most of the VEGF-expressing cells were GFAP-positive astrocytes. In MeHg-exposed rats, intravenous administration of anti-VEGF neutralizing antibody mildly reduced the rate of observed hind-limb crossing signs, a standard motor functional sign considered an expression of limb ataxia in animal models of MeHg intoxication. Thus, we demonstrated that MeHg induces BBB damage by upregulating VEGF expression in the BBB in vivo. In our study, upregulation of VEGF was detected only in the cerebellum. The BBB of the cerebellum is thought to be more vulnerable than that of the cerebrum because the expression levels of tight junction proteins and *P*-glycoprotein, one of the barrier proteins, are lower in the cerebellum than in the cerebrum [109,110]. Moreover, vascular permeability reportedly is increased more strongly in the cerebellum than in the cerebrum in nonphysiological conditions, such as inflammation [109,110,111,112]. The selective damage, hemorrhage, and edema in the cerebellum as seen in the brains of patients may be explained in part by the specificity of VEGF expression in the cerebellum. Increased VEGF expression causes vascular hyperpermeability, which exacerbates several disease conditions [113,114,115]. Administration of anti-VEGF neutralizing antibody suppressed the deteriorative effect in our experiment [108] as well as in these conditions [113,114].

To date, there are no established therapies to treat mercury poisoning. Chelating therapy, which accelerates the excretion of mercury [116,117], is recommended, particularly in the early phase of mercury exposure. However, evidence for the beneficial effect of chelation therapy is limited, and chelating therapy occasionally induces adverse effects, including sudden cardiac death, because chelators also increase the excretion of essential metals. In addition, in the USA, inappropriate use of chelating agents promoted by alternative medicine societies without essential clinical examination for mercury exposure has raised concern. Although there might be beneficial effects in some cases, chelating agents should be used with caution. While chelating therapy alone is not sufficient to improve symptoms, it is possible that combinations with other therapeutic approaches, such as antioxidant or anti-VEGF neutralizing therapy, can reduce the toxicity of mercury. Combination therapy aimed at maintaining the BBB, suppressing oxidative stress, and chelation may have a synergic and thus better effect.

## 6. Summary

- MeHg causes severe damage to the CNS. MeHg penetrates the BBB into the brain parenchyma via active transport systems, mainly LAT1, on membranes of endothelial cells.
- Growing evidence suggests that even low-level MeHg exposure can induce endothelial dysfunction and increase the risk of cardiovascular disease.
- The most widely reported mechanism of MeHg toxicity is oxidative stress and its consequences, such as neuroinflammation.
- VEGF upregulation is observed after MeHg exposure in vitro and in vivo. The selective damage in the cerebellum after MeHg exposure may be explained in part by the specificity of VEGF expression in the cerebellum.
- BBB dysfunction has been suggested by studies on in vitro and in vivo models of MeHg intoxication, and therefore, maintaining the BBB may represent a promising therapeutic strategy for the treatment of MeHg intoxication.

## Abbreviations

MeHg	Methylmercury
BBB	Blood–brain barrier
CNS	Central nervous system
LAT1	L-type amino acid transporter 1
VEGF	Vascular endothelial growth factor
AQP4	Aquaporin-4
RECA-1	Rat endothelial cell antigen-1
IgG	Immunoglobulin G
